# Doula Services Within a Healthy Start Program: Increasing Access for an Underserved Population

**DOI:** 10.1007/s10995-017-2402-0

**Published:** 2017-12-02

**Authors:** Mary-Powel Thomas, Gabriela Ammann, Ellen Brazier, Philip Noyes, Aletha Maybank

**Affiliations:** 1 NYC Department of Health and Mental Hygiene, New York, NY, USA; 2 Institute for Implementation Science in Population Health, CUNY School of Public Health, New York, NY, USA

**Keywords:** Doula support, Health equity, Disparities, Pregnancy and birth outcomes, Birth inequities, Maternal health, Race, Women of color, Healthy Start

## Abstract

**Purpose:**

Women of color in the United States, particularly in high-poverty neighborhoods, experience high rates of poor birth outcomes, including cesarean section, preterm birth, low birthweight, and infant mortality. Doula care has been linked to improvements in many perinatal outcomes, but women of color and low-income women often face barriers in accessing doula support.

**Description:**

To address this issue, the New York City Department of Health and Mental Hygiene’s Healthy Start Brooklyn introduced the By My Side Birth Support Program in 2010. The goal was to complement other maternal home-visiting programs by providing doula support during labor and birth, along with prenatal and postpartum visits. Between 2010 and 2015, 489 infants were born to women enrolled in the program.

**Assessment:**

Data indicate that By My Side is a promising model of support for Healthy Start projects nationwide. Compared to the project area, program participants had lower rates of preterm birth (6.3 vs. 12.4%, p < 0.001) and low birthweight (6.5 vs. 11.1%, p = 0.001); however, rates of cesarean birth did not differ significantly (33.5 vs. 36.9%, p = 0.122). Further research is needed to explore possible reasons for this finding, and to examine the influence of doula support on birth outcomes among populations with high rates of chronic disease and stressors such as poverty, racism, and exposure to violence. However, feedback from participants indicates that doula support is highly valued and helps give women a voice in consequential childbirth decisions.

**Conclusion:**

Available evidence suggests that doula services may be an important component of an effort to address birth inequities.

## Significance

Women of color and those in areas of high poverty face persistent inequities in birth outcomes. Doula support during pregnancy and childbirth is associated with improvements in many outcomes, including lower rates of cesarean section and preterm birth, higher rates of breastfeeding initiation, and increased satisfaction with the birth experience. However, socioeconomic, cultural, structural, and systemic factors limit access to doula care and its benefits. Increasing access may help address inequities in pregnancy outcomes for Black women and women in high-poverty neighborhoods. Experience from Healthy Start Brooklyn suggests that doula support is a promising complement to other home-visiting programs.

## Purpose

In the United States, women of color—particularly those living in areas of high poverty—experience disproportionately high rates of poor birth outcomes, including cesarean delivery, preterm birth, low birthweight, and infant mortality (Roth and Henley 2012; Hamilton et al. 2015; Infant Mortality 2016). These disparities in birth outcomes likely result from various factors, including the disproportionate burden of preexisting health conditions and risks faced by Black and Latina^1^ women (Braveman et al. 2011), as well as chronic stressors such as poverty and structural, interpersonal, and internalized racism (Latendresse 2009; Rosenthal and Lobel 2011). Recent research shows even after adjusting for co-morbidities, hospitals serving predominantly Black populations have higher rates of severe maternal morbidity than hospitals serving predominantly White populations (Howell et al. 2016). The disparities in infant and maternal health outcomes reflect the stark challenges women of color face in obtaining high-quality care (Roth and Henley 2012; Gruber et al. 2013).

Continuous support during labor is a potential means of mitigating disparities in perinatal outcomes. Labor support has been linked to fewer instrumental deliveries, shorter labors, higher APGAR scores among newborns, and increased satisfaction with the birth experience (Hodnett et al. 2013; Gruber et al. 2013). One source of labor support is a doula, a trained childbirth professional who provides emotional, physical, and informational support to women during labor, delivery, and the immediate postpartum period. Supporting women and families in their decision-making during childbirth and early parenting is a central aspect of doula care (DONA International 2014). Evidence suggests that doula support provided prenatally and during childbirth may both lower maternal stress and enhance women’s self-efficacy regarding their pregnancy and ability to manage labor (Hodnett et al. 2013; Gruber et al. 2013; Kozhimannil et al. 2013). In addition, a doula’s respect for the autonomy of the client and emphasis on positive goal-setting during prenatal visits have been shown to increase women’s empowerment and confidence regarding their ability to influence their own pregnancy outcomes (Gruber et al. 2013; Gentry et al. 2010; Breedlove 2005). Psychosocial support from a doula during labor may also attenuate maternal stress, thereby contributing to better birth outcomes (Gruber et al. 2013; Christopher and Simpson 2014).

Studies conducted with low-income Black and Latina women in programs that included prenatal, labor, and postpartum support have shown lower rates of cesarean birth, increased breastfeeding initiation, and longer duration of breastfeeding (Kozhimannil et al. 2013; Gruber et al. 2013; Harris et al. 2012; Nommsen-Rivers et al. 2009; Mottl-Santiago et al. 2008). A study in Minnesota found that Medicaid recipients who received prenatal doula support had lower rates of preterm birth than other Medicaid recipients in the region (4.7 vs. 6.3%) (Kozhimannil et al. 2016). A South African study showed that low-income women in an urban community hospital who received non-medical companionship during labor and birth had decreased rates of depression at 6 weeks postpartum (Wolman et al. 1993).

Despite the benefits of doula support, doula care is underutilized, particularly among low-income women and women of color (Kozhimannil et al. 2014). Barriers include lack of information about services, lack of available services, and cost. A national survey found that women whose delivery was covered by Medicaid were almost 50% less likely to know about doula care than women who were privately insured (DeClerq et al. 2013). Lantz et al. found that most doulas in the United States are White, upper-middle-class women, which has implications for the availability of doula services in other communities (Lantz et al. 2005). Finally, doula support is not routinely covered by health insurance and thus is beyond the means of many low-income women (Kozhimannil et al. 2013).

To address inequities in birth outcomes, Healthy Start Brooklyn (HSB) offers free doula services in Black and Latino neighborhoods that have been historically deprived of resources. Launched in 2010, the By My Side Birth Support Program makes doula care available to women who meet income eligibility requirements for the Women, Infants, and Children nutrition program (WIC). Project strategies and birth outcomes among program clients to date are described below.

## Description

The By My Side Birth Support Program (BMS) serves pregnant women living in the neighborhoods of Brownsville, East New York, Bedford-Stuyvesant, and Bushwick—communities with a disproportionately high burden of poor health. Nearly one-third (31.3%) of adults are obese, 35.0% have been told they have high blood pressure, and 15.1% have been told they have diabetes (DOHMH 2014). More than 30% of the population lives below the federal poverty line (U.S. Census Bureau 2014), and the project catchment area includes neighborhoods with the highest levels of community violence in New York City (King et al. 2015). These and other social determinants likely contribute to disparities in pregnancy outcomes, including an infant-mortality rate as much as 76% higher than the NYC average (7.4 deaths per 1000 live births in East New York vs. 4.2 citywide), as well as preterm birth and low birthweight rates that are the highest in the city (14% preterm birth in Brownsville vs. 8.8% citywide, and 11.6% low birthweight in Brownsville vs. 8.8% citywide) (Li et al. 2016).

Healthy Start Brooklyn was launched in 2001 by New York City’s Department of Health and Mental Hygiene (DOHMH), with funding from the Health Resources and Services Administration of the U.S. Department of Health and Human Services. The program focused on non-Latina Black women, the group with the highest infant-mortality rate in the program area. One key part of the initiative was a free childbirth-education series, taught by a local doula and Lamaze instructor. Over time, she noticed that many participants reported not having reliable support for their labor and delivery. Some women’s partners were unable to take time off during the workday; others needed to rely on their partner or their own mother to care for older children rather than providing labor support. To respond to this need, the instructor began matching her students with volunteer doulas. In 2010, HSB formalized the process with the launch of By My Side, hiring certified doulas as independent subcontractors and paying them a professional rate to support women and their partners during pregnancy, childbirth, and the postpartum period.

BMS combines aspects of private doula practice and community-based programs to provide social support during pregnancy, labor and delivery, and the postnatal period. Private doulas typically provide one to two prenatal home visits, support during labor and delivery, and one postpartum home visit. Community-based doula programs typically provide many more visits throughout pregnancy and may continue services for an entire year postpartum.

BMS doulas conduct three prenatal home visits, covering the traditional doula curriculum (prenatal care, stages of labor, birth preferences, communicating with care providers), as well as screening for depression, food insecurity, intimate partner violence, and medical risk factors, and making referrals to services as needed. Because this case-management function is outside the typical scope of doula work, BMS provides additional training, as well as an extensive resource guide of services in and near the project area. Multiple visits give the doula time to discuss childbirth topics in a relaxed setting, when the woman has time to think about the information, do additional research, and decide what her preferences for labor may be. The relationship that develops during these visits also improves continuity of care during labor, especially for women who have never met any of their obstetrical-care providers before arriving at the hospital.

In addition to support during and immediately after the birth, BMS doulas often provide assistance to pregnant women and their families in navigating the hospital environment during labor, facilitating communication with the medical staff. They make a follow-up visit within 2 days of the birth and another at 2 weeks postpartum. In early 2015 two additional home visits were added, at 2 and 6 months postpartum. During the four postpartum visits, doulas support breastfeeding, assess client and infant risks, and provide counseling on safe-sleep practices, positive parenting, and reproductive life planning. Home visits last 1–2 hours each.

Merging the private and the community-based models of care provided HSB the flexibility to develop a doula program that complemented its existing home-visitation services, a Nurse-Family Partnership program and a Healthy Families America program, both of which provided support from pregnancy through the first few years of a child’s life, but not during the birth itself. BMS addresses this gap by providing participants with continuous support during labor and birth, either as the only service that women receive or in addition to other home-visitation services.

BMS currently subcontracts with 12 doulas, four of whom have been with the program since 2010. Staff longevity has contributed to programmatic stability; the experienced doulas provide support to those new to the program, building on lessons learned and sharing their expertise. For example, each new doula who joins the program is paired with an experienced BMS doula, who mentors her in classic doula care, as well as best practices in supporting clients who may be navigating multiple stressors. Two of the newest doulas to join the program are themselves former clients.

## Assessment

BMS doulas collect the same client data as other HSB home-visiting programs, including characteristics of program participants, plus labor and delivery data. Between 2010 and 2015, BMS served more than 560 women, and 489 infants were born to program clients; 84.7% of these births were attended by doulas. The average age of these clients at the time of enrollment was 27.1 years (standard deviation of ± 6.2 years). Attrition has been relatively low: 83.2% of the infants’ mothers remained in the program through graduation (defined as completing at least two postpartum visits).

Demographic characteristics of births from 2010 to 2015 are summarized in Table 1, along with birth-record data for all infants born in the program area from 2010 to 2014, the most recent data available. Chi-squared tests were used to compare differences, using p < 0.05 as the cut-off value for statistical significance. Table 1 highlights important differences between program births and resident births overall, representing both higher and lower social risk factors for BMS participants. A greater proportion of BMS participants are non-Latina Black (84 vs. 59%, p < 0.001), have Medicaid or another public insurance (90 vs. 79%, p < 0.001), and are enrolled in WIC (82 vs. 74%, p < 0.001), compared with overall rates in the program area. At the same time, however, BMS participants have higher levels of education (58% with more than a high school degree vs. 43%, p < 0.001), and they were more likely to begin prenatal care in their first trimester of pregnancy (80 vs. 64%, p < 0.001). Medical risk factors are not reported here; however, BMS does not exclude anyone based on risk, even those who are having a planned cesarean delivery.

**Table 1:** Selected characteristics of By My Side program participants compared to residents overall in project area, by live births

	By My Side (N = 489)		Project area* (N = 34,912)		p value**
	2010–2015		2010–2014		
	#	%	#	%	
Race/ethnicity					
Non-Latina Black	410	84	20,740	59	p < 0.001
Non-Latina White, Asian Pacific Islander, Latina, other, unknown	79	16	14,172	41	
Age					
Mean age	27	–	27.7	–	
Insurance					
Medicaid/public insurance	438	90	27,441	79	p < 0.001
Private insurance, no insurance, unknown	51	10	7471	21	
WIC					
Enrolled in WIC	402	82	25,950	74	p < 0.001
Timing of first prenatal care					
First trimester (1–90 days)	390	80	22,107	64	p < 0.001
Late/no/unknown prenatal care	99	20	12,805	37	
Education					
More than high school	285	58	14,894	43	p < 0.001
High school or less	204	42	19,750	57	

*Data from NYC DOHMH Department of Vital Statistics: birth outcomes for zip codes 11207, 11208, 11212, 11216, 11221, and 11233 in 2010–2014

**p values for Chi Square tests calculated with SAS (version 9.4)

Table 2 provides a comparison of outcomes for BMS births against those of the program area. The Cesarean-section rate is statistically similar across both groups (33.5 vs. 36.9%, p = 0.122); however, BMS participants have significantly lower rates of preterm birth (6.3 vs. 12.4%, p < 0.001) and low birthweight (6.5 vs. 11.1%, p = 0.001).

**Table 2:** Birth outcomes for By My Side program participants compared to residents overall in project area, by live births

	By My Side (N = 489)	Project area* (N = 34,912)	p value**
	2010–2015	2010–2014	
	N (%)	N (%)	
Cesarean section	164 (33.5%)	12,894 (36.9%)	p = 0.122
Preterm birth (< 37 weeks)	31 (6.3%)	4319 (12.4%)	p < 0.001
Low birthweight (< 2500 g)	32 (6.5%)	3882 (11.1%)	p = 0.001

*Data from NYC DOHMH Department of Vital Statistics: birth outcomes for zip codes 11207, 11208, 11212, 11216, 11221, and 11233 in 2010–2014

**p value for exact Fishers test (1-sided) at 95% confidence interval (1-tailed) calculated with SAS (version 9.4)

In addition to collecting quantitative data, BMS conducts periodic follow-up telephone interviews with former clients, including both graduates and dropouts, using a semi-structured questionnaire. To minimize possible sources of bias, interviews are conducted by someone who does not know the client or the doula. The follow-up interviews reveal high levels of satisfaction with the program: Of 244 clients surveyed between July 2010 and January 2015, 95.9% said they would recommend the program or use it in a future pregnancy, and 94.3% said they were “well-matched” with their doula. Table 3 provides examples of comments by participants about how their doula made a difference during their labor and delivery.

**Table 3:** BMS program-participant comments on the ways their doula made a difference during labor and delivery

• “She didn’t push anything on me. She gave me information, and then I chose what I wanted”
• “When [the hospital staff] would say I needed certain things, she let me know that it was my decision if I wanted it or not, and that I didn’t have to do anything I didn’t want to. She let me know that I had a voice and a choice”
• “I would’ve had no one there; it was just me and her. If it wasn’t for her, maybe I wouldn’t even get through it, because she really helped a lot”
• “She told me step by step what my options were while we were in the delivery room. The doctors and nurses try to force you to do things that you don’t have to do, just because it’s better for their practice, or their hospital, or their insurance, or whatever the case may be. But it’s not always good for you. You do it anyway because you trust the doctor. But I trusted my doula more, and she gave all my options on the table so I could decide what was best for me, you know?”
• “She showed me she believed in me”

## Conclusion

Healthy Start Brooklyn’s By My Side Birth Support Program appears to be a promising model for providing support to women living in communities with persistent disparities in birth outcomes. Client feedback and relatively low rates of attrition indicate that doula support is highly valued by BMS clients and helps women have a voice in consequential perinatal health decisions. Results from the BMS program are consistent with findings from other studies of the benefits of doula care, particularly for women in historically disadvantaged communities who face a range of financial, informational, and access barriers to these services.

Although it is not possible to determine a causal relationship between program exposure and birth outcomes, because of self-selection and other potential sources of bias, preterm birth and low birthweight are lower among program clients compared to the project area overall. However, rates of cesarean birth are not significantly lower than background rates for the program area, and more research is needed to explore the possible reasons for this finding. In particular, it would be useful to examine how routine hospital practices in the program area, such as confining a woman to bed during labor, may affect doula support and limit the influence a doula can have on mode of delivery. It would also be helpful to assess how and in what circumstances doulas affect hospital practice. More research is also needed on the influence of doula support on birth outcomes among populations experiencing high rates of chronic diseases, such as obesity, hypertension, and diabetes, along with the interlinked challenges of pervasive poverty, community violence, and structural racism.

Doula programs staffed by volunteers and/or funded by grants such as Healthy Start have been established to address inequities in birth support. However, the sustainability of these initiatives over time is unclear, and funding limitations preclude the provision of services to all women who would benefit from them. Recognizing these challenges, a growing body of stakeholders is calling for Medicaid programs and private insurers to cover doula support (Kozhimannil et al. 2013). Two states, Minnesota and Oregon, recently passed legislation to provide Medicaid reimbursement for doulas (Kozhimannil et al. 2014). In New York, HSB co-founded the New York Coalition for Doula Access, a group of doulas, community-based doula administrators, other Healthy Start grantees, health-department staff, and others across the state that is working with medical professionals to address barriers that doulas face in hospitals. The coalition has also begun exploring potential pathways to third-party reimbursement, which would offer a means of making doula care financially viable. This may increase access to doula care in neighborhoods with high poverty, where many pregnant women are not able to pay for these services. However, it would be important for state Medicaid offices to set their rates high enough to provide a living wage for doulas, so as not to perpetuate systems of inequity.

The By My Side Birth Support Program experience shows that low-income Black and Latina women in Brooklyn neighborhoods are currently underserved by doula support and that when enrolled in these services, they benefit from increased emotional, physical, informational, and social support provided during pregnancy, childbirth, and the postpartum period. Providing access to free doula support in communities of color and high-poverty neighborhoods may be one important component of the effort to address persistent inequities in birth outcomes.

## Footnotes

1 For the purposes of this publication, “Latino/a” includes persons of Hispanic, Latino, and Spanish origin.
